# Resiliency of the Iranian healthcare facilities against the Covid-19 pandemic: challenges and solutions

**DOI:** 10.1186/s12913-023-09180-6

**Published:** 2023-03-01

**Authors:** Farahnaz Ezzati, Ali Mohammad Mosadeghrad, Ebrahim Jaafaripooyan

**Affiliations:** Department of Health Management and Economics, School of Public Health, Tehran University of Medical Sciences, Tehran, Iran

**Keywords:** Resilience, Health Organization, Challenges, COVID-19

## Abstract

**Background:**

Health care facilities are responsible for preventing and controlling diseases and must be resilient enough to deal with crises. The Iranian health care facilities have faced challenges in managing COVID-19 pandemic. The purpose of this study was to identify the challenges faced by the Iranian health care facilities during the Covid-19 epidemic and to provide solutions.

**Methods:**

This qualitative study was conducted with a phenomenological approach and using semi-structured interviews with 59 healthcare policy makers, managers, and employees, and medical university faculty members. The participants were selected through purposive and snowball sampling. Thematic analysis was used to analyze the data.

**Results:**

Overall, 43 challenges to the resilience of health care facilities during the Covid-19 pandemic were identified and grouped into 8 themes (i.e., leadership and management, planning, organizational culture, organizational learning, employee management, customer management, resource management, and process management. The most important resilience challenges were: fragmented management system; poor leadership; incompatible health network structure; lack of a national holistic plan; poor case detection; insufficient resources; inefficient information system; negative attitude of managers and employee; organizational inertia; failure to build on lessons learned from crises; low workforce preparedness; lack of community-based management; and improper monitoring and evaluation. Managers should use community-based, evidence-based, and integrated management to build health system resilience against COVID-19, have sufficient knowledge and experience to organize operations, use appropriate and effective coordination models, develop a creative and participatory culture, reengineer processes, and provide necessary resources.

**Conclusion:**

The Iranian health care facilities face challenges that prevent them from becoming resilient, responsive, and efficient in managing COVID-19. Policy makers and managers should increase the resilience of health care facilities to shocks and crises by using the suggested measures.

## Background

The health system includes individuals, groups and organizations that perform the functions of policy making, financing, generating resources and providing healthcare services to restore, promote, and maintain public health [1]. Leadership and governance, financing, health workforce, equipment and supplies, health information systems, and service delivery are the six building blocks to achieve the health system goals [2]. Without investing in the health system and strengthening its components, its goals cannot be achieved sustainably [3]. Strengthening the health system and its building blocks is essential to achieve the ultimate goals of improved health, financial risk protection and responsiveness.

Iran is a middle-income country in Middle East with a population of 84 million people [4] and GDP per capita of international $ 15791 [5]. The Iranian health system is a mixed of public, private, and non-government organizations involving in financing and delivery of healthcare services. The Ministry of Health and Medical Education is responsible for financing, policy making, planning, and controlling healthcare organizations at the national level. Medical universities have the same role at the provincial level. The district health network delivers primary healthcare (PHC) services and the hospital network delivers specialized healthcare services [6].

The primary goal of the health system is to promote and maintain the health and well-being of the population while preventing and controlling diseases. Therefore, the health system should ensure that everyone has access to quality and affordable health care services. Health care facilities must be responsive and meet the needs of the population. In addition, they must respond and adapt to epidemiological, economic, political, and social shocks such as disasters, wars, economic crises and epidemic diseases. They must be ready for crises in order to properly respond to them and provide the health services needed by the people during the crisis.

Health system resilience is defined as “the ability, capability and capacity of the health system to predict, prevent, prepare, absorb, adapt and transform when exposed to shocks and stresses and deliver routine health services continuously during the crisis management” [7]. Resilient health care facilities constantly evolve in response to the external environment. They have flexible employees, adaptable supply chains, and agile organizational structures, and are able to adapt to sudden changes in the environment [8].

Experiences from the 2009 H1N1 pandemic, the 2014-2016 Ebola outbreak in West Africa, and the 2015-2016 Zika virus epidemic in Latin America and Southeast Asia have shown that countries with fragile health systems have difficulty coping with acute shocks. In the wake of the Ebola outbreak in Guinea, Liberia, and Sierra Leone, the World Health Organization (WHO) focused on strengthening the health system, and encouraged policy makers and senior managers to enhance the resilience of health care facilities [9]. Building up the resilience of health care facilities plays a key role in health care delivery, especially in times of crisis and catastrophe. Strategies should thus be developed to enable health care facilities to respond quickly and effectively to acute, sudden, and severe shocks and crises [10].

The COVID-19 pandemic is the most critical global health crisis in the last century with its rapid spread and associated disease burden and mortality. COVID-19 was declared a pandemic by WHO on March 11, 2020. The Covid-19 virus infected 642.9 million and killed about 6.6 million people worldwide by December 8, 2022 [11]. Iran reported the first confirmed cases of COVID-19 on February 19, 2020. Almost 7.6 million people were infected and 145,000 people were died by December 8, 2022 as a result of Covid-19 [11]

Health care facilities are extremely vulnerable to the severity of this disease. Studies have shown that the Iranian health system has been moderately resilient against COVID-19 [12, 13]. The Iranian health care facilities are ill-prepared to deal with these disasters and crises and face many challenges such as political and structural instability, high top management turnover, financial constraints, too much responsibility with too little authority, and the changing expectations of patients and the public. Therefore, in addition to coping with sudden acute shocks, these organizations must also respond to chronic challenges. Therefore, policy makers and managers of health care facilities must take the necessary steps to create resilient organizations that can provide quality and safe services to the population in spite of these shocks and challenges.

The present study aimed to identify the challenges to the resilience of health care facilities in dealing with COVID-19. The results can provide valuable insights to healthcare managers, policy makers, and experts to better manage this crisis.

## Methods

This is a qualitative study with an interpretive phenomenological approach. Qualitative studies are suitable for exploring and explaining topics about which we know little [14]. In an interpretive phenomenological research, the researcher collects rich in-depth data to better interpret the findings and generate new knowledge and ideas [15]. Interpretive phenomenological method is suitable for identifying, examining, describing, explaining and interpreting phenomena, facts, events, processes, activities and concepts about which we have little knowledge.

Interpretive phenomenology includes three stages of primary understanding of the research subject, conducting a study and collecting information, and focusing and reflecting on the data [16]. For this purpose, before starting the research, the researcher must acquire enough knowledge and information about the research subject in order to collect deep data and information during the research and to have a better interpretation of the obtained findings during the data analysis. As a result, the researcher produces new ideas and knowledge with a complete understanding of the research topic.

Data were collected from April to September 2021 through in-depth and semi-structured interviews with 59 participants including policy makers (15), managers and employees of the healthcare facilities (27) as well as faculty members of medical universities (17). The majority of the participants were male (88.1%), married (96.6%) and had a doctorate degree (56%). About 96% of the interviewees had more than 10 years of work experience (Table 1).

**Table 1:** The demographic characteristics of the research participants

Demographic Characteristic	Frequency	Percentage
Gender		
Female	7	11.9
Male	52	88.1
Age		
30-40 yrs.	6	10.2
41-50 yrs.	28	47.5
51-60 yrs.	23	38.9
> 60 yrs.	2	3.4
Marital Status		
Married	57	96.6
Single	2	3.4
Education		
Master’s Degree	11	18.6
General practitioner (GP)	8	13.5
Ph.D.	33	56
Medical consultant	7	11.9
Experience		
5-9 years	2	3.4
10-15 years	7	11.9
16-20 years	9	15.2
21-25 years	17	28.8
26-30 years	18	30.5
> 30 years	6	10.2

An interview guide was developed based on the objectives of the study. Purposive and snowball sampling methods were used to select the participants. Most interviews were conducted online due to the COVID-19 pandemic. The interview guide was sent to the participants before the interview. All interviews were recorded after obtaining the consent from the respondents. Interviews lasted about 50 minutes on average (maximum 90 minutes and minimum 30 minutes) and continued until saturation.

Braun and Clarke’s 6-step thematic analysis procedure was used to analyze the data [17]. The interviews were transcribed in Microsoft Word 2016 and were reviewed several times. Initial codes related to the resilience challenges of Iranian health care facilities were identified and extracted from the transcripts. Similar codes were grouped into subthemes and then into main themes. These themes were reviewed several times and combined and adjusted as needed to generate a thematic map of the analysis. Then, the main themes and subthemes were labeled and defined. MAXQDA 20 software was used for data analysis. In this article, the quotations were identified with the letter P.

Several measures were taken to enhance the trustworthiness (i.e., credibility, authenticity, transferability, dependability, and confirmability) of the findings, including pilot interviews, maximum variation sampling, prolonged engagement in the field, examination of the subject from different angles, collection of as much information and evidence as possible, triangulation, member checking, peer debriefing, and description of the research environment and phases.

The ethical considerations of the research included: obtaining ethical approval from Department of Research at Tehran University of Medical Sciences; obtaining informed consent from the participants; allowing the participants to withdraw from the research at any time; respecting the autonomy of the participants; asking for permission to record interviews; maintaining confidentiality of the data and protecting the anonymity of the interviewees; and ensuring the objectivity of the researchers in all stages of data collection, analysis, and processing.

## Results

Overall, 43 challenges to the resilience of health care facilities during the Covid-19 pandemic were identified and grouped into 8 themes: leadership and management; planning; organizational culture; organizational learning; employee management; customer management; resource management; and process management (Table 2).

**Table 2:** Resilience of health care facilities in COVID-19: challenges and solutions

Themes	Challenges	Solutions
Leadership and management	• Fragmented management system • Person-centered decision-making • Inadequate coordination • Lack of a unified command structure • Lack of a preventive approach • Insufficient management commitment • Incompetent managers • Poor leadership • Incompatible network structure • Poor organizing	• Development of incident command system (ICS) protocols • Community-based crisis management • Evidence-based management • Effective operational command • • Good governance • Enhancing managers’ risk perception • Developing a resilient mindset in students • Internal and external coordination • Prioritizing prevention at the national level
Planning	• Lack of a national plan • Poor case detection program • Lack of strategic and operational plans • Lack of using key indicators • Lack of different scenarios	• Appointing competent managers • Holding practical courses • Documenting and building on past experiences • Changing the management structure • Developing strategic and operational plans • Reforming the command-control structure and coordination in the country • Formulating a comprehensive plan and a national crisis management structure
Organizational culture	• Negative attitude of managers and employee • Organizational resistance • Organizational inertia • Poor resilience culture • Insufficient teamwork	• Promoting the right mindset in managers and employee • Understanding resilience • Changing managers’ attitudes • Creating everyday flexibility • Encouraging creative and innovative ideas • Effective conflict management
Organizational learning	• Inadequate training of managers and employee • Inadequate exercises • Failure to measure effectiveness • Failure to build on experiences and lessons learned	• Regular theoretical and practical training • Systematic academic education Documenting and building on experiences
Employee management	• Ineffective employee selection and recruitment process • Insufficient awareness among employee • Low employee competence and skill • Unpreparedness of employee • Insufficient support for employee	• Continuous training • Changing the behavior of managers and employee • Training to recognize the work process • Employee empowerment • Financial incentives • Increasing employee motivation and satisfaction • Providing family support for employee
Customer management	• Lack of community-based management • Inadequate training; Lack of public empowerment • Lack of effective communication between the health system and the public • Lack of belief in public participation in health programs • Declining social capital • Insufficient involvement of non-governmental organizations	• Motivating people • Giving people responsibility • Building trust • Effective communication with people and non-governmental organizations • Making use of people’s capacities • Transparent job descriptions • International communications • Conducting exercises and drills • Childhood education • Effective communication • Effective organization of people • Community-based management
Resource management	• Inadequate resources (especially financial and physical resources) • Inefficient information system • Economic instability • Lack of a central portal • Low capability of the integrated health system	Increasing public health expenditure per capita • Working toward lifting sanctions • Capacity building across the country • Improving economic stability • Having a unified information system • Educating employees • Developing a central portal for data management • Upgrading integrated health system programs
Process management	• Poor structure of the health system • Lack of a system for managing work processes • Insufficient evaluation and monitoring	• Self-organization at the time of crisis • Processes reengineering • Eliminating bureaucracy • Monitoring and evaluation systems • Promoting everyday resilience • Self-evaluation

### Leadership and management

Fragmented management system, person-centered decision-making, lack of coordination, failure to establish a unified command structure, lack of a preventive approach to healthcare, lack of managers’ commitment, poor management, ineffective leadership, incompatibility of the network structure, and poor organizing were highlighted as key leadership and management challenge of health care facilities resilience.

Having an integrated management system is an important factor in taking advantage of the potential of other agencies. A department manager in a university said: “*Crisis management requires an integrated management system. This issue hasn’t received enough attention. Not only should the health system and the Ministry of Health function properly, but they also need to coordinate with and get support from the Red Crescent, municipalities, and other organizations*” (P4). Evidence-based management and building on the global experience was one way of addressing this challenge: “*We are awful in leadership and management. What is evidence-based would be right. Our current actions and decisions are not evidence-based*” (P10).

Lack of intra-sectoral and inter-sectoral coordination is a major challenge to COVID-19 leadership and management as it increases the likelihood of delays and wasted time in projects, and programs. Organizations must coordinate the activities of their members and establish good relationships with other organizations to achieve their goals. The participants believed that a lack of well-defined job descriptions in health facilities was contributing to the lack of coordination in the health system. The director of the disaster management department of one of the universities commented that, “*there is a lack of coordination within the university’s department of health and between the department and ministries involved in healthcare such as the Ministry of Health, the Ministry of Agriculture, the Ministry of Education, etc*.” (P12).

The lack of an incident command system and a crisis response system is a major challenge to the resilience of health care facilities. The participants considered the incident command system to be an important factor in health system resilience: “*The crisis response system is different from a regular response system. In a crisis, our response system must be directed toward incident command so that we can use our capacities to the fullest*” (P12). Some health care facilities adopted a management restructuring strategy to address the lack of a crisis response system: “*As soon as a state of emergency was declared, decisions were made at the national level to assign new responsibilities to us. This didn’t happen. Everyone went their own way as their regular operations was being disrupted by the pandemic*” (P10). Similarly, a COVID-19 control managers argued: “*We do not have unity of command in the governorate. All agencies should work together, the responsibilities of all departments should be clear, but everyone is acting on their own*” (P15). Coordination and implementation of the program was an important challenge. “*Job descriptions are often poorly defined in critical situations. Now that the national COVID headquarters has been established and job descriptions have been defined for different departments, we have problems in implementation. Planning and implementation should go hand in han*d” (P8).

According to the participants, coordination between committees and knowledge of each committee’s responsibilities were important. “*Now during the COVID crisis, the Minister of Health has set up several scientific associations and committees; five to six to be exact, none of which knows the other at all. Is that really necessary? Shouldn’t we have a single entity in charge, and if not, shouldn’t these associations and committees communicate and coordinate better?*” (P18). Some managers highlighted the role of governance in this regard: “*Just because a committee is established doesn’t mean that people carry out their duties properly. If there are rogue elements that disrupt the program, the health sector should coordinate with the municipalities. Therefore, not only should every department affiliated with the Ministry of Health get involved in this matter, but each should also identify the external agencies they should get help from in order to do their job. Now, if we have an authority delegated by the highest levels of government, it can also define the responsibilities of the Ministry of Health*” (P18). The same manager suggested having a coordination model as a way to improve the situation: “*First, we have to have plans, and secondly, these plans should specify a coordination model, and by that, I mean both internal and external coordination*” (P18).

Lack of a preventive approach to health care was another major challenge to the resilience of health care facilities in controlling COVID-19. For example, the head of an academic department at one of the universities said: “*One of the weaknesses of health care facilities in Iran is their missions and goals. For them, ‘prevention before cure’ is just a slogan. Another problem is that hospitals are viewed as the custodian of public health rather than the health system, and naturally missions, goals and strategic planning, if there are any, are developed with that mindset*” (P1). Managers’ lack of belief in prevention was one of the problems raised by the participants. “*In the country, treatment has always been a priority. Public health is prevention, but because we don’t believe in it, it hasn’t been a priority. For example, currently the budgets that should be allocated to public health are mostly allocated to treatment. That’s because if a problem occurs in a hospital during treatment, it’ll make headlines, but if someone has an intestinal disease due to lack of access to basic sanitation facilities, nobody will hear about it*” (P41).

The proposed solution is to prioritize prevention within the health system. A faculty member at one of the universities said: “*They we run our system isn’t cost-effective. Why is it that we don’t have enough budget to control non-communicable diseases even though it’s a priority? We don’t have the same GDP as many developed countries, but spent as much as they do on health care. So, the problem isn’t that we don’t have money, it’s that we don’t spend it well. Resilience means spending on prevention. 90% of the people require education for prevention, but we spend 90% of the budget in hospitals. We wait for people to get sick, to have a stroke, and get cancer instead of trying to prevent diseases. In other words, we’re increasingly shifting toward medicalization*” (P26). Another faculty member stated: “We can increase our resilience by focusing on prevention, because it decreases the number of hospital visits in the long run and healthcare burden in the long run, or at the very least reduces acute care costs” (p27).

Many of the participants believed that the commitment and attitude of the managers of health care facilities is an important factor in the resilience of the health system against COVID-19. Self-centeredness and partisanship prevent managers from developing a national vision. The director of disaster risk reduction at one of the universities said: “*Many managers across the country are driven by in-group favoritism. The role of disaster managers isn’t defined anywhere in the COVID landscape and we’re not included at all in some high-level meetings. Self-centeredness and partisanship shape managers’ views, not what’s good for the nation. They talk a lot, but do little*” (P3). Managers should have high risk perception and understanding and consider disaster indicators in their planning. “*The biggest problem in our country is that managers don’t want to hear the truth. Our most important challenge is their low understanding of risks and their total disregard for disaster indicators*” (P3). Lack of managerial commitment can affect the commitment of employees and their attitude toward resilience. One faculty member commented: “*When health care managers and officials take the issue seriously, the employees follow suit. Lack of managerial commitment could be the bane of resilience at the time of crisis*” (P24).

Selecting managers that lack knowledge and expertise can be an important challenge to the resilience of health care facilities. One university administrator stated: “*For me, most of the problems in this area are rooted in the lack of management knowledge and expertise; many managers know little about resilience and therefore have the wrong attitude*” (P43). The director of disaster management at one of the universities described strong and effective management as an important factor in boosting resilience: “*Majority of problems are caused by mismanagement. An ineffective manager will definitely have a negative impact on the employee and the organization. Our health system suffers from poor management, which happens for various reasons. For example, many managers don’t receive the necessary training and have to learn through experience, which can be detrimental for the organization and its processes. In addition, there are no templates or checklists for appointing managers. In many instances, candidates don’t have the necessary qualifications and less qualified ones are selected*” (P54).

Leadership is crucial to successful achievement of organizational resilience. Leadership is the ability to influence, persuade, and mobilize individuals to achieve a goal. A university faculty member said: “*By leadership, I mean the power of influence that exists in all organizations. We don’t have leaders in the health system in the classical sense of the word. Without proper leadership, resources won’t be allocated efficiently. Why do we have about 19 parallel health insurance companies? I attribute it to the lack of unified leadership in the health system*” (P26). A faculty member highlighted the importance of strengthening the leadership role as a means for achieving greater resilience. “*I can’t stress the role of leadership enough. Leaders have followers who accept their authority and, in many cases, trust them despite the fact that the conditions aren’t favorable. My suggestion is that we promote leaderism instead of managerialism in this area*” (P22).

Incompatibility of the management system and the structure of the health network as well as the principles of primary health care has caused challenges to health system resilience. One deputy director of the Ministry of Health stated: “*I don’t think our management system is at all compatible with the structure of our health network or our needs*” (P10). One faculty member suggested the application of primary health care principles as a solution: “*If we apply the principles of PHC, we really don’t have to do anything new. It enables us to become more resilient, which is also in line with resistance economy. We don’t need new foundations; we just have to put existing theories into practice, to implement the four principles of PHC: equitable distribution; community participation; intersectoral coordination; and appropriate technology*” (P26). The inability to organize a response was one of the problems as noted by one university faculty member: “*Our weakness is that we don’t have a plan in place, we don’t organize a crisis response. We haven’t designed and established an operational command center*” (P12).

### Planning

The participants considered factors such as lack of a national plan, poor case detection program, lack of strategic and operational plans, lack of key indicators, and lack of contingencies for different scenarios as important barriers to the resilience of health care facilities against COVID-19.

The lack of a national response was cited as a reason for the failure to follow the guidelines. The director of pre-hospital emergency medicine at one of the universities said: “*We don’t have a national response that is based on the conditions of the country and the boundaries between departments and organizations*” (P14). Having a coherent national plan facilitates the adoption of programs by other agencies and the implementation of guidelines. “*We don’t have a coherent national plan for responding to disasters and no national framework; as a result, the plans and guidelines developed by the Ministry of Health don’t translate well into the world outside the ministry and aren’t implemented as intended*” (P14).

Some managers argued that it is not enough to just have a plan and that it is important to understand the roles it entails and how to implement it. One deputy director of the Ministry of Health said: “*The problem is that you may go to Dr. X and ask him, ‘what plan do you have for disasters?’ They give you a very nice plan, but the Network Management Center doesn’t know about it; the Public Health Office doesn’t know about it. Therefore, just because a plan is formulated well and adheres to standards doesn’t mean that all parts of the system understand and accept their role and how to carry it out. Only when this is addressed can we say that there is a plan beyond what’s on the paper*” (P18). A university administrator offered the development of strategic plans as a solution: “*When we have strategic planning and a strategy, even our everyday decisions and operations should be in line with that strategy*” (P14).

Many of the participants considered the lack of operational and strategic plans as an important barrier to the fight against coronavirus. The director of the educational department of one of the universities said: “*The health system needs operational and strategic plans that include contingencies for different scenarios, so that if plan A fails, there’s always a plan B*” (P1). The head of the risk reduction unit of one of the universities highlighted impulsive decisions and lack of pre-crisis planning as important barriers. “*There should be pre-crisis plans for different scenarios and roles should be clearly defined with future adjustments if needed*” (P6).

In this regard, one faculty member stated: “*You see, it was around December that the first cases of COVID-19 were reported in China. We knew that the pandemic would reach our country. As soon as the first COVID cases were reported in Qom, there was a shortage of alcohol and masks the next day. Why didn’t we anticipate it beforehand?*” (P26) Similarly, another faculty member commented: “*China built two hospitals in only a few days, which means that all of this had already been planned. This capacity doesn’t exist in our country and our health system isn’t resilient*” (P31). The director of pre-hospital emergency medicine at one of the universities suggested the following solutions to these challenges: “*Reforming the command-control and coordination structures in the country, developing a comprehensive plan and a national crisis management structure, having a disaster response plan, and upgrading management practices*” (P14).

### Organizational culture

Negative attitude of managers and employee, organizational resistance, organizational inertia, low resilience culture, and lack of teamwork were among the cultural challenges to the resilience of health care facilities against COVID-19. Some of the participants believed that negative attitude has become especially challenging during this crisis. The disaster management director of one of the universities said: “*The cultural discourse is complex. There is the concept of KAP: knowledge, attitude, and practices. We may not have a problem in terms of knowledge right now, but people don’t have the right attitude to respond to disasters. When managers are warned of imminent disasters and their response is ‘let’s see what happens’ or ‘what God wills, will happen’, we have a serious cultural problem that is not conducive to resilience and preparedness. People have become complacent as a result of our culture, so both our cultural problems are both general and organizational*.” He continued by underlining routine seeking and organizational inertia as major challenges: “*Unfortunately, our organizations suffer from inertia, routine seeking behavior, and silence. COVID-19 has provided a window of opportunity for us to overcome these deficiencies and build on our capacities*” (P12).

Promoting the right beliefs in managers and employee was another solution as noted by the director of disaster risk reduction at one of the universities stated: “*The most important factor in resilience is capacity building, which can be a cultural issue. And by culture, I mean beliefs that shape behavior. Unless we promote the right beliefs, things will never change. This should be our ultimate goal*” (P3). According to the disaster management director of one of the universities, “*the solution is to change the attitude of the managers and promote more positive attitudes and beliefs*” (P54).

Resistance to change among employees leads to low resilience. One faculty member noted: “*Our health workers are not up-to-date. They lack the latest information, capabilities, and knowledge, and often resist change*” (P5). The head of the risk reduction unit of one of the universities suggest everyday flexibility as a way of reducing organizational resistance: “*Everything we do faces resistance. We must encourage everyday flexibility, introduce it gradually and embed it into every unit over time. It must be one of our values*” (P6).

Lack of resilience culture in the organization is an important barrier to organizational resilience. “*I believe that resilience isn’t ingrained in our organizations yet; that is, if we want to achieve resilience on a large scale, as in a big organization, there should be an understanding of what it is and what it entails. If organizations don’t understand resilience, they won’t be able to implement it, they won’t understand its definitions and concepts, they will suffer from entropy, they will become obsolete, and this is to the detriment of the organization. Of course, models are a good thing. They have their place, but I think a deeper discussion is needed*” (P22).

Some interviewees believed that a lack of team spirit has undermined the resilience of health systems. Regarding the absence of a culture of empathy and teamwork, one deputy director of the Ministry of Health said: “*Our most important weakness in the health system is lack of support, empathy, and teamwork. We have organizations with defined structures that must communicate and coordinate with each other. But inside each organization, there is little support and teamwork between different units. The climate is competitive. We don’t have a team culture and little teamwork in scientific, administrative, or medical areas*” (P34). A faculty member discussed leadership as a means for promoting resilience culture: “*We must promote the culture of resilience. Of course, this can’t be done in a week. It takes time, but introducing leadership can help in the promotion of resilience culture*” (P22).

### Organizational learning

Participants considered lack of common understanding of organizational learning, lack of vision to promote resilience, lack of measurement of resilience, inadequate training of managers and employee, inadequate exercises, failure to measure effectiveness, and failure to build on experiences and lessons learned as challenges to health system resilience against COVID-19: “*Our pre-hospital emergency system and our accident system are more familiar with resilience and the literature than our health system. Before anything else, the health system must prepare its own employee*” (P1). A senior university administrator said: "*Our problem is that we do not have a clear vision for learning resilience in the organization, that is why a common understanding has not been created in our organization*)"p2).

A technical assistant commented: “*We aren’t familiar with the resilience discourse. This work has just begun. Given its importance, it’s necessary to enhance the understanding and awareness of this issue to be able to boost resilience in health care facilities. It can be as simple as the workshops we held for operational planning and crisis management*” (P8). The COVID control director of one of the universities stated that theoretical and practical training is the key to crisis preparedness: “*Theoretical and practical courses can be organized to increase awareness and preparedness of the personnel so that they can respond effectively to crisis situations. We often teach theory, but when crisis happens, we run around like a headless chicken*” (P15). Systematic academic education was another area for promotion of resilience as highlighted by one of the interviewees: “*Resilience should be taught systematically in academic studies. There should even be a special unit in the Ministry of Health or the Health and Food Security Council to plan for it and supervise it*” (P8). The participants mentioned "*the Resilience should become a culture, which means that we should give training and change behavior, if these steps do not happen, it is difficult to implement*” )P50(.

COVID control director at one of the universities believed that impracticality of training is an important barrier to planning. “*Our problem is lack of practical experience. We participate in seminars many times, we attend crisis committee meetings, but when they ask us to write a scenario for a magnitude 6 earthquake, we really struggle*” (P13). Holding practical courses was a solution to solve this challenge: “*In fact, theoretical and practical courses can raise awareness and enhance the preparedness of personnel, which enables them to should act properly in times of crisis*” (P15). One faculty member noted: "*To improve organizational resilience, the amount of resilience learning should be measured and the amount of resilience learning should be influenced in the career advancement of employees*”(p5). Another director described documenting and building on experiences as an effective solution to this challenge: “*Documenting (experiences) is important. It is important to know what went wrong and what worked, and now we’re learning how to disseminate information. If we used experiences from COVID-19 and similar crises, we would’ve responded more effectively*” (P7).

### Employee management

Employees are the most important resources of an organization that must be developed and empowered to strive toward achieving organizational goals. Factors such as ineffective employee selection and recruitment process, lack of awareness among the employee, low employee competence and skill, unpreparedness of staff, and lack of support for staff are identified as key challenges in the face of COVID-19. One of the interviewees described ineffective employee selection and recruitment process as a resilience challenge in this domain: “*Service delivery starts with health workers, but given the growing urbanization, it’s not appropriate to recruit health workers with a high school diploma. The literacy rate is on the rise, highly educated people in villages don’t like to receive services from these less-educated health workers*” (P14).

The participants cited the unpreparedness of health care employee as a major challenge to dealing with the crisis: “*The Ministry of Health was not prepared for the current situation [COVID-19 crisis]. People’s lives were at stake, and the response was to prioritize hospitals. The system relied on ICUs and specialty and subspecialty protocols, and now the main concern of the Ministry of Health is to reduce it mortality rate. The protocols have been based on intensive care, which is extremely costly. After months, the health system needs reform, both for COVID response, which is not clear how long it will last, and for post-COVID recovery*” (P1). Another manager said: “*Our employees aren’t prepared. We don’t work seriously on this issue. As a middle manager, I didn’t think we would have a crisis. I didn’t know how to work in a crisis situation. I had to do everything myself. I didn’t have an organized protocol and chart. We weren’t prepared to be resilient*” (P11).

One of the senior managers argued that the solution is to change the behavior of managers and employee: “*A change of attitude and work behavior is needed at all three levels*” (P2). Interviewees considered the lack of employee support and protection as an important challenge. “*In special circumstances, like the COVID-19 crisis, it is necessary to provide support to employee to improve retention, which contributes to the resilience of health care facilities*” (P13).

The participants mentioned the lack of incentives and employee dissatisfaction as the reasons for their reduced participation. The director of one of the health departments said: “*Look, as a member of the organization, I should know what my mission is and what my responsibilities are in relation to resilience; I should feel confident that senior managers support me, financially or otherwise, so that I can do my job effectively when crisis happens; I should have enough motivation to work with enthusiasm and passion, but this is not the case right now. Motivation, compensation, and fatigue are very important in times of crisis*” (P11). Another manager stated: “*When employees aren’t satisfied, we can’t expect them to show up to work when an accident occurs. Participation in decision-making is also important to employees and affects their satisfaction*” (P4). The COVID-19 management director of one of the universities suggested support for employees and their families and financial incentives as a solution: “*Increasing the motivation of the employee is the first step. They also have families in times of crisis. Family support, good overtime payment, and empowerment are very important in this regard*” (P15).

### Customer management

In the customer management domain, the participants highlighted challenges such as lack of community-based management, inadequate training, lack of public empowerment, lack of effective communication between the health system and the public, lack of belief in public participation in health programs, declining social capital, and lack of involvement of non-governmental organizations (NGOs). Some of the interviewees argued that organizing people is very important for crisis management, but it is lacking. The director of pre-hospital emergency medicine at one of the universities said: “*We should work toward more community-based crisis management. There are very few community organizers and they do not work effectively, including the Red Crescent. I think the most important role of the Red Crescent is to educate and organize people to take action during disasters. To prepare people, we’ve limited ourselves to a series of individual training, but little has been done for organizing people to enable them to manage a neighborhood in times of crisis*” (P14). Another manager highlighted the lack of community-based management as a reason for low public participation: “*We should always support communities, financial, human, physical, and spiritual support. To manage a crisis, we must communicate with people and promote community-based management during crises and disasters. Unfortunately, many organizations don’t approach this properly and have failed to coordinate people during COVID*” (P12).

The interviewees considered the lack of public awareness as factor influencing resilience against the crisis. A senior university administrator said: “*People should know what to do during crisis. Right now [during COVID], people should take care of many health issues themselves and raising awareness has become more important than ever*” (P2). Another director drew attention to inadequate training during the COVID-19 crisis: “*People should be educated on this. COVID showed that much of our training is ineffective. Even now, some people still don’t wear masks. We should change the way we train people*” (P15). He also considered holding exercises and drills to increase public awareness: “*Public awareness is very important. There have been some exercises, but not enough. We need continuous exercises and drills*” (P15). In addition, childhood education at home and school were suggested as the best solution: “*Educating people about disasters and resilience should start in kindergartens and schools; a 60-year-old may not learn or not remember. Unfortunately, we didn’t do anything until the crisis*” (P15).

Empowering people is critical to increasing the resilience of the population and the health system. A faculty member said: “*Let me give you an example. 10 years ago, if you needed to make any financial transaction, you had to go to the bank. But now you can do almost everything yourself. That’s empowerment! The health system hasn’t been able to achieve that. If people can take care of much of their health issues themselves that increases the resilience of communities as well as the health system. Health spending will decrease. The integrated health system provides a good platform for achieving this goal*” (P26).

Lack of effective communication between the health systems was another key challenge: “*The strategy of the Ministry of Health has been leaning toward self-care. Self-care is very effective for prevention of diseases. But the ministry hasn’t communicated well with people. If people know what’s good for their health and well-being, they will do it*” (P2). The same participant argued that giving people responsibility and autonomy in managing their healthcare as an effective way of improving relationships with people: “*For example, employees have a password and can log into the SIB system, but the general public doesn’t have that ability*” (P2).

From the perspective of some of the managers, the lack of belief in public participation in health programs was a serious challenge to health system resilience: “*Our weakness is the lack of belief in the need for people’s participation in health care and this view is systemic*” (P16). He suggested building trust as a strategy to overcome this challenge: “*It’s important to have the knowledge and skills necessary to involve people in health issues. Trust is a key factor in resilience, and that also requires skill and a methodical, systematic approach*” (P16).

Lack of government transparency reduces social capital, and this is an important challenge to the resilience of health care facilities. “*I think the declining spiritual and social capital is also a threat. The relationship between people and the government is important. When people lose trust in the government and the relationship between the people deteriorates, social capital decreases. It’s not only driven by economic factors, but also by the transparency of government officials*” (P8).

Lack of proper communication with people and NGOs is one of the factors reducing resilience. One deputy director of the Ministry of Health said: “*Lack of coordination and communication with people and NGOs is a major challenge. We need their help for the implementation of programs. So, it is very important to gain the trust of these organizations. They take a lot of the burden away from the government. Communication with local representatives is also important*” (P17).

Transparency of job descriptions in the health system and their legitimacy for the people can increase resilience: “*Building trust is very important. If the health team had established good relationship with the people, they wouldn’t behave like this during this crisis and would’ve been more cooperative. The role of the health system should be clear to people. In places where minorities lives, we must gain their trust as well*” (P17).

### Resource management

Lack of resources, especially financial and physical resources, inefficient information system, sanctions, economic instability, lack of a central portal, and low capability of the integrated health system were the main challenges in the domain of resource management. From the participants’ point of view, inadequate funding was an important barrier to an effective response to the crisis. “*As a risk reduction manager, I submitted several requests for equipment, but none of the items was provided due to lack of funding*” (P3). Another manager described the problem of lack of resources as follows: “*There is no budget item for disasters, even though Iran is one of the top 10 disaster-prone countries in the world. We can’t suddenly decide to build a field hospital with no planning or budget. In the existing hospital, operating room employee are being transferred to COVID wards*” (P13). The shortage of equipment was described as a major challenge in dealing with the COVID-19 crisis: “*Another critical issue is the lack of equipment and supplies, especially in laboratories*” (P1). Moreover, increasing public health expenditure per capita was suggested as an effective solution to the problem of lack of funding for health care facilities: “*It is important to increase public health expenditure per capita. Currently, the treatment sector eats up a large part of our budget. In the COVID crisis, the first line is covered by public health experts and health workers. It’s not just doctors and nurses who are involved*” (P11).

Many of the participants considered sanctions as a factor in the lack of resources and a major challenge to health system resilience: “*Sanctions have hindered the supply of equipment, medicine, test kits, etc*.” (P1). “*Lack of financial resources can be a threat, which is becoming more and more alarming as the crisis grows in scale. Sanctions have made it especially difficult for us to purchase necessary equipment and supplies*” (P17). Most interviewees suggested that working toward lifting the sanctions and capacity building within the country are effective solutions to these challenges: “*We must use all the tools and capacities at our disposal and build on them*” (P1).

Some of the participants believed that there is no single information system in the country. “*We have difficulty communicating information about coronavirus. We do not have a single information system. People should be kept up-to-date with the latest information and news about the pandemic and other healthcare issues*” (P13). In this study, misinformation was identified as an important impediment: “*It is very important for the health system to have a strong presence in social media. It is also crucial to use all capacities to prevent the spread of misinformation and rumors through different types of media*” (P1). One faculty member stated: “*Decision makers should have the right information to make the right decisions. There are now 50 different stories about COVID and vaccines. Some say the vaccine works, some say it’s harmful. This can cause a great deal of confusion in the society*” (P27).

One of the deficiencies of the health information system was the inability of the integrated health system to be controlled by service recipients, which could reduce the number of hospital visits during the COVID-19 crisis: “*Cyberspace has grown exponentially, but we still haven’t been able to exploit this potential effectively. For example, people can only receive services in person; it used to be registered in logs and now it’s registered in the system*” (P14).

Economic instability is an important barrier to resilience against the COVID-19 crisis. “*A major challenge is that our country is going through a lot of changes, both externally and internally. External changes, like the value of dollar, affects every decision. Our plans and budgets are also affected. There are also internal factors that contribute to economic instability in the country, such that we can’t even plan for a month from now. Managers are trying their best, but without stability, day-to-day management is the only thing that can do*” (P14). According to one of the faculty members, “*The country’s economic instability causes insanity in all sectors. The main challenge that managers face is that their plans constantly change*” (P5).

### Process management

The participants highlighted factors such as poor structure of the health system, lack of a system for managing work processes, and lack of supervision and monitoring as challenges to health system resilience in the process management domain. Many of the participants considered the outdated structure of the health system as a major challenge during crisis: “*Our health structure is very old and not suitable for new services. The system is not resilient and rejects change” (P10). The health system structure is not well-suited to times of crisis, which acts as a barrier to organizational resilient. “We have a structural problem. The structure isn’t designed for times of crisis. Our management system should be like a military system*” (P13).

Poor *management of working processes was another challenge in dealing with the pandemic. “Our PHC structure is not in line with demographic changes and processes are just like they were in the 1980s*” (P14). A lack of self-organization during crises was also highlighted: “*Systems should be self-organizing. We should identify opportunities and capacities. When disaster occurs, each organization as an ecosystem should be able to maintain itself and rebound. That’s what resilience is all about*” (P4)

Reengineering structures and processes and eliminating bureaucracy are effective solutions for dealing with the above challenges: “*We seem to be stuck in an old and obsolete administrative system in the country. It should be reengineered. Bureaucracy is out of control! We can’t achieve much as long as we have systematic corruption*” (P13). “*We should review our plans. For example, the vaccination plan that we’re working on, should it be localized based on the specific conditions of the country or not? Also, the current COVID process can help US identify strengths, weakness, opportunities, and threats. That is, our post-COVID health system shouldn’t be anything like what we have now; this pandemic can be a learning experience and a planning exercise. We had SARS in 2014; COVID appeared in 2019. There may be another outbreak or pandemic in a few years, so the health system should be able to build on these experiences to reengineer its own structure, processes, and goals*” (P1).

Periodic monitoring of the resilience program is essential. According to a senior university administrator, “*we have weaknesses in all aspects of resilience, including the environment, processes, and procedures. There is no periodic monitoring and review of either employee empowerment or process standardization*” (P7). To overcome this challenge, the participants suggested “*researching and developing a monitoring and evaluation system for resilience*” (P8). Moreover, one faculty member proposed everyday resilience and periodic monitoring as effective solutions: “*There should be a written, systematic program for resilience and that it should be part of everyday discussions. It should … be structured in such a way that all programs have periodic monitoring and oversight, both for the existence of crises and the resilience of the network structure*” (P5). Another director described organizational self-assessment as a solution to the lack of monitoring and evaluation: “*All organizations should conduct self-assessments to identify their strengths and weaknesses so that they can plan to help themselves and communities become more resilient*” (P17).

**Fig. 1:**
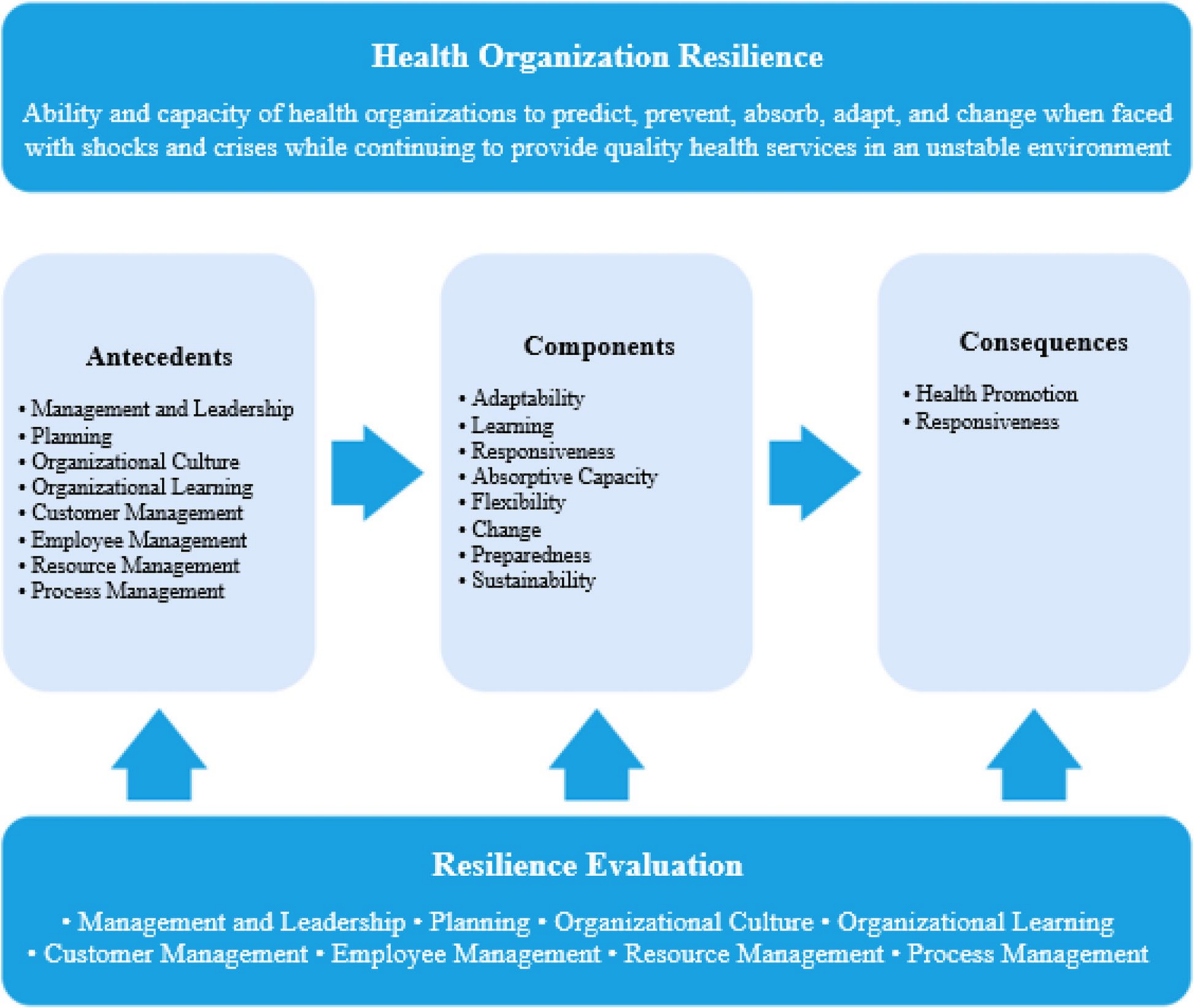
A Conceptual model of health organization resilience

Finally, a conceptual model for resilience of health organizations was designed (Fig. 1). This model consisted of eight antecedents (i.e., management and leadership, planning, organizational culture, organizational learning, customer management, employee management, resource management and process management). Adaptability, learning, responsiveness, absorptive capacity, flexibility, change, preparedness, and sustainability are the main elements of health care facilities’ resilience. Health promotion and responsiveness are the main two consequences of healthcare facilities’ resilience.

## Discussion and conclusion

The purpose of this study was to identify the challenges to the resilience of health care facilities against COVID-19. Poor leadership and management, poor planning, inappropriate organizational culture, lack of attention to organizational learning, and ineffective management of employees, customers, resources, and work processes were identified as the main challenges. Finally, a conceptual model of resilience of health organizations was developed.

The success or failure of organizations in crises depends to a large extent on their managers. Poor leadership and management is an important barrier to strengthening the resilience of health care facilities [18]. Senior managers’ commitment and support is essential for the resilience of health care facilities as it will increase participation and commitment of employee in implementing resilience plans. It is essential to develop resilience thinking and improve risk perception in health care managers and employees.

Crisis management requires an integrated management system to streamline the process of managing organizational operations. Establishing an integrated command structure between the Ministry of Health and other organizations involved can increase coordination. Decentralized management and delegating authority to local levels can strengthen the responsibility and accountability of health care facilities. Using a decentralized structure and empowering local health care facilities will enable them to respond more quickly to environmental challenges and mobilize local resources to respond to the crisis as compared to top-down hierarchical structures [19]. Clear communication channels must be established between the Ministry of Health and other sectors of society so that policies can be translated into action.

Senior managers should develop the necessary guidelines and standards for different departments to create a safe and stable environment for employees and customers. Managers need to develop their leadership abilities and skills. The role of leadership in motivating employees, creating a culture of voluntary service, and supporting employees is crucial to participation of employees in the implementation of programs. Effective leaders must articulate the role of health care facilities in preventing, identifying, and effectively addressing public health threats. One of the key characteristics of effective leadership is its ability to demonstrate the importance of making the health system more resilient in situations where it is difficult to convince policy makers of the importance of prevention and preparedness. Leaders must show that investing in health systems is vital for emergency situations.

Leadership is a key factor in organizational resilience. In South Africa, health care facilities with committed and strong leaders were more resilient [20]. Health care managers must create a vision for employees in the event of a crisis, build trust, empower, train and motivate them, increase their commitment, and provide them with the necessary resources. Participatory leadership is more appropriate for complex adaptive organizations such as health care facilities and can increase their resilience [21].

Early in the COVID-19 pandemic, it became clear that rapid implementation of public health measures and health system operations depended on effective coordination at all levels and between different departments. Coordination of activities between the government and stakeholders is essential [22]. This means ensuring effective collaboration between sectors, different levels of government, governmental and non-governmental stakeholders, and international bodies. For instance, the Health Alert and Emergency Coordination Centre was created in Spain as a mechanism for coordination between the national and regional governments [23]. Similar channels were developed during the 2003 SARS outbreak among specific Asian countries and were quickly activated during the COVID-19 pandemic [24]. Significant successes in combating the COVID-19 pandemic in some countries such as Australia or South Korea have been attributed in part to integrated coordination between ministries, departments, and agencies [8].

Planning is one of the most important tasks of managers. Without a proper plan, it is impossible to consistently achieve organizational goals [25]. Assessment of the status quo is a prerequisite in planning and crucial to effective performance in dynamic environments [26]. Health organizations in Singapore during the SARS outbreak in 2003 simulated possible scenarios and developed management plans accordingly [24].

Preparedness consists of three elements: resources (alternative resources), functions (planning and determining crisis stages), and training of individuals and leaders. The Ebola crisis in West Africa has highlighted the importance of epidemiological surveillance as part of an alert system in controlling the spread of the disease [22]. Obviously, preparing for challenges requires an understanding of the risks and threats as well as awareness of possible solutions and organizational requirements. Policy makers and senior executives should hold regular meetings to review strategic and operational plans related to organizational resilience. Regular monitoring of programs prepares the organization for a crisis and facilitates rapid post-crisis recovery and reconstruction.

It is necessary to develop a strategic plan, involve key stakeholders, and use evidence to monitor and evaluate the performance of health care facilities. Managers need to be able to identify the challenges that threaten the organization in order to develop an appropriate plan to respond to those challenges. This capacity must be acquired during the normal state of the health system, since when crisis happens, more time and resources are spent responding to the crisis and its negative impacts.

Organizational culture is a set of shared assumptions, beliefs, values, and norms that define the way in which an organization conducts its business and achieves its goals [27]. Health care managers must make changes in their organizational culture in order to increase commitment and resilience, everyday flexibility, and collective mentality [28]. Improving communication between managers, employee and patients, promoting collaboration, and training the employee will provide the necessary cultural context. Managers and employees must believe in continuous resilience against shocks and view it as a dynamic goal to strengthen the health system. In addition, the culture of society should be strengthened in terms of social responsibility, and people should reduce the spread of Covid-19 by following health protocols, using masks appropriately, physical distancing and minimizing contact with others.

Learning lessons from previous shocks is necessary for identifying effective responses to similar crises [29]. Health care managers and employees should build on lessons learned, both nationally and internationally and across systems, in order to increase their organizations’ resilience. Without learning and evaluating past experiences, little progress can be made toward preparing for similar situations [30]. Transforming health care facilities into learning organizations is useful for enhancing resilience.

Organizational learning play an important role in the resilience of health care facilities. It is “the ability of an organization as a whole to discover errors and correct them, as well as change the knowledge and values of the organization so that new problem solving skills and new capacity for work will be created” [31]. Previous studies have also emphasized the importance of training and empowering managers and employees in organizational resilience [9, 29]. Employees training has a significant effect on reducing their resistance. Employees should receive the necessary training and believe that developing and implementing a resilience plan is necessary and useful for the organization. Employee training and development should be part of the organizational learning process in health care facilities. A culture that promotes learning and adaptation can build resilience [9].

Motivated and committed employees participate more and play an important role in the success of the organization [32]. The attitudes of employees towards challenges and their ability to learn from experiences in crises and be creative and innovative are key elements of a resilient organizational culture [29]. Having a strong, flexible and motivated workforce is an important element of preparedness that allows organizations to adapt to any shock. Health care employees are at the forefront of response to crises, especially disease outbreaks, and without proper support and management, they will suffer the most and will lose motivation [9].

Lack of government transparency reduces social capital, and this is an important challenge to the resilience of health care facilities. Transparency of job descriptions and roles in the health system, public trust, open communication, and legitimacy of the health system are important for resilience. If the health system establishes proper, transparent relationship with the people, people will be more cooperative in the times of crisis.

Community-centered and people-centered management during disasters and raising public awareness should receive serious attention from health care managers. Affected people are the first responders in emergencies. Experience has shown that the most successful disaster risk reduction programs have been those implemented with the participation of the people or by the people themselves. Identifying key actors in communities and integrating indigenous and local knowledge with scientific evidence can contribute to the development of resilient communities. A resilient community knows its capabilities and vulnerabilities, either through learning or experience and traditional and local wisdom [33].

Resources are essential for service delivery by health care facilities. In the event of a crisis, having sufficient and well-distributed resources can save time for capacity building and provide the necessary resilience. In contrast, being shocked by existing shortages may widen gaps in access to care and undermine the response [9, 29]. The lack of resources and equipment, limited testing ability and inadequate surge capacity were listed the main weaknesses of Africa’s health system to deal with COVID- 19 [34]. Health systems are primarily funded from general taxation and social insurance contributions. Shocks often affect these sources of funding. The key to ensuring a resilient health system is to create financing mechanisms that are impervious to shocks, accumulate reserves, and create automated stabilizers activate in response to shocks [9].

An important factor in resilience is to ensure that there is enough money in the health system and that these resources are readily available in the event of a crisis. In response to COVID-19, many countries have injected additional funds into their health systems. For instance, Lithuania quickly allocated a significant amount of additional funds to its health system [9]. The government in Singapore allocated 42.4 billion USD (12.2% of gross domestic product) to protect businesses and people who lost their income during COVID-19 epidemics [24]. Similarly, the Spanish central government allocated €2800 million to support the health system and protect businesses [23]. To withstand the crisis, financial resources must be quickly directed to where they are needed. In some countries, this is done through the accumulation of national reserves, while others have laws that direct financial resources toward the health system. In fact, a flexible response may require the government to temporarily increase health care budgets and lower fees so that patients can use the services they need [35].

Using the right information system during crises plays a crucial role in increasing resilience. Information is a key component of timely and effective adaptation to challenges in the health system. Previous studies have also found the use of an appropriate information system to be effective for achieving greater organizational resilience [29, 30]. A recurring feature of resilient systems is continuous data collection to improve preparedness and response to both long-term changes and shocks. It is important to use data to predict, prepare, and respond effectively to changes, make evidence-based decisions, provide regular monitoring and evaluation, and perform system analysis [30].

Process reengineering, and elimination of bureaucracy are essential for better management of the COVID-19 pandemic. Process management should be one of the main areas of focus of health care facilities. Having good resources and structures is a necessary condition to strengthen the resilience of healthcare organizations. In addition, working processes should be standardized and optimized to provide quality and safe services during crises [36]. Furthermore, healthcare organizations should have a system for monitoring and evaluating resilience programs. Managers should develop resilience performance indicators for health care facilities. The performance of healthcare disaster risk management programs should be measured and corrective measures should be taken [37].

### Limitations

A conceptual model for health organization resilience was developed in this study which needs to be confirmed empirically. The research findings may not be generalized to developed countries, which may have different structure and culture.

## Conclusions

Iranian health care facilities face challenges that prevent them from becoming resilient, responsive, and efficient. It is imperative for health policy makers and managers to take necessary measures to ensure the sustained delivery of high-quality and safe care in spite of these shocks and challenges, and the results of the present research may serve as a useful guide. A strong primary health care system is a prerequisite for strengthening the Iranian health system. Strengthening management and leadership, formulating disaster risk reduction plans, improving organizational structures and cultures, and managing employees, customers, resources and processes effectively make the health system resilient and sustainable.

## Data Availability

The data that support the findings of this study can be obtained from the Iranian Ministry of Health, but restrictions apply to the availability of these data, which were licensed for use in the present study and are not publicly available. However, data can be obtained from the authors upon reasonable request and with the permission of the Iranian Ministry of Health.

## Abbreviations

ICS: Incident Command System
MOHME: Ministry of Health and Medical Education
